# A randomised trial of the effect of postal reminders on attendance for breast screening

**DOI:** 10.1038/bjc.2015.451

**Published:** 2016-01-19

**Authors:** Prue C Allgood, Anthony J Maxwell, Sue Hudson, Judith Offman, Gillian Hutchison, Cathryn Beattie, Raquel Tuano-Donnelly, Anthony Threlfall, Tina Summersgill, Lesley Bellis, Collette Robinson, Samantha Heaton, Julietta Patnick, Stephen W Duffy

**Affiliations:** 1 Centre for Cancer Prevention, Wolfson Institute for Cancer Prevention, Charterhouse Square, London EC1M 6BQ, UK; 2 Nightingale Centre and Genesis Prevention Centre, University Hospital of South Manchester, Southmoor Road, Wythenshawe, Manchester, M23 9QZ, UK; 3 Peel and Shriek Consulting, 182 Highbury Hill, London, N5 1AU, UK; 4 Bolton Hospital NHS Foundation Trust, Minerva Road, Farnworth, Greater Manchester, BL4 0JR, UK; 5 Royal Liverpool University Hospital, Prescot Street, Liverpool, Merseyside, L7 8XP, UK; 6 Wrightington, Wigan and Leigh NHS Foundation Trust, Appley Bridge, Wigan, Lancashire, WN6 9EP, UK; 7 Theorize Ltd, 134 Slade Lane, Levenshulme, Manchester, M19 2AQ, UK; 8 Visiting Professor in Cancer Screening, University of Oxford, Cancer Epidemiology Unit, Richard Doll Building, Roosevelt Drive, Oxford, OX3 7LF, UK

**Keywords:** breast screening attendance, randomised trial, postal reminders

## Abstract

**Background:**

Some women make an informed choice not to attend breast screening, whereas others may have forgotten about the appointment. We report on a randomised trial that investigates whether a reminder letter affects attendance.

**Methods:**

Women scheduled for a breast screening appointment were randomised to either receive a reminder letter a few days before their breast screening appointment in addition to the standard invitation letter (intervention) or not (control). The primary outcome was attendance within 30 days of the first offered appointment. Secondary outcomes were attendance within 90 and 180 days.

**Results:**

In all, 11 383 (49.9%) women were randomised to the intervention and 11 445 (50.1%) to the control. In the intervention arm, 7759 (68.2%) attended within 30 days of the first offered appointment compared with 7349 (64.2%) in the control arm. This difference was significant (*P*<0.001). The odds ratio (OR) (95% confidence interval) for the primary end point was 1.19 (1.13–1.26). This was not significantly affected by age, socioeconomic status or type of screen (prevalent or incident). Secondary endpoint analyses supported these results. Results did differ, however, between the different centres studied.

**Conclusions:**

This study found that postal reminders increase breast screening uptake, and could be practicable to implement in the NHS Breast Screening Programme.

The effect of a screening programme on breast cancer mortality depends, in part, upon high participation rates. The service has a duty to allow the women invited to make up their own mind as to whether to take up the offer of screening, but it is incumbent on the provider to acknowledge that the service is offered because screening has been found to prevent deaths from breast cancer (Marmot *et al*, 2013). This independent review was undertaken because of the recent and highly publicised criticism of breast screening (Gøtzche and Nielsen, 2011), although the concerns have been shown to be largely unwarranted (Duffy *et al*, 2012). Although the Marmot review concluded that the benefits of breast cancer screening far outweighed the harms, there is no doubt that this could have had a negative effect on breast screening attendance. Attending regularly is important as cancers detected symptomatically in women who have been invited to breast screening but have not attended tend to be characterised by poor survival (Lawrence *et al*, 2009).

There are a number of interventions that can improve participation in cancer screening programmes. These include telephone, face-to-face, postal and text reminders (Fleming *et al*, 2012; Offman *et al*, 2014; Shankleman *et al*, 2014; Kerrison *et al*, 2015), GP endorsement (Hewitson *et al*, 2011), second timed appointments for non-attenders (Stead *et al*, 1998) and flexibility as regards appointment times (Offman *et al*, 2013).

In the UK's National Health Service Breast Screening Programme (NHSBSP), attendance rates for screening have slowly declined nationally over the past few years. In 2004–2005, 74.6% of invited women aged 50–70 years attended, but this had fallen to 72.2% by 2012–2013. The fall was more marked for women in the first (prevalent) round of screening, where attendance across the United Kingdom fell to 68.1%, compared with 71.0% in 2005–2006, and is now below the national minimum standard of 70% (NHS Cancer Screening Programmes, 2007; NHS, 2013).

Some women who do not attend screening may have made an informed decision not to attend, but others will have intended to go to the appointment but simply forgot about it and therefore failed to attend. Here we report on a study to estimate the effect on participation of a postal reminder sent a few days before a woman's appointment.

## Materials and methods

We conducted a randomised controlled trial in three screening units in the North-West of England: Bolton, Bury and Rochdale (referred to as Bolton), Wigan and Liverpool. Women were randomised 1 : 1 by each woman's screening reference number (Sx number) by a simple coin toss at the commencement of the study to determine whether the intervention arm (to receive the reminder letter) would be women with odd- or even-numbered Sx numbers. The Sx number is a unique number allocated to women when they first come to breast screening and which they keep throughout their screening life. There was no reason to believe that a difference existed between the characteristics of women with odd and even Sx numbers as there is no systematic practice with regard to the SX number allocated. Appointment and attendance data were downloaded from the National Breast Screening System database.

All women who were due to be invited to their regular three yearly or first breast screening appointment within the time period of this study received an invitation letter giving ∼3 weeks notice of a dated and timed screening appointment, following normal screening programme procedures. Approximately 2 weeks later, a letter reminding them of this appointment was generated and sent to the women in the intervention arm of the trial (Supplementary Material). They received this letter a few days before their original screening appointment. Women in the control arm did not receive this reminder letter. Care was taken to ensure that the reminder contained accurate information and was non-coercive in tone. The important role of the reminder was to draw the invitee's attention to their imminent screening appointment. The letter is given in the Supplementary Material.

Women were excluded from this study for the following reasons:

Women outside the age range 50–70 years. Although the eligible age range has recently been extended to women aged 47–73 years, it has not yet been fully implemented in all screening units.
Women under surveillance for a higher risk of breast cancer due, for example, to a family history of breast cancer.
Women who were recalled early because of either a suspicious finding on a mammogram or a technical reason.
Women who had cancelled their appointment before generation of the ‘reminder date', and who did not rebook at a later date (this exclusion was to ensure non-coercion of those who had firmly decided not to attend).

Overall attendance in the two arms was compared using descriptive tables of numbers and percentages attending, with formal statistical inference using logistic regression. Data were analysed using the STATA 12 software statistical package (StataCorp, 2011), reporting deviance, *χ*^2^ tests, odds ratios (OR) and 95% confidence intervals. Subgroup analyses were performed for prevalent and incident rounds, previous non-attenders, patient age and socioeconomic status, which was measured by the index of multiple deprivation (IMD) (Communities and Local Government, 2010). Four end points were used:

Attendance report 1 (primary end point):

- Attendance within 30 days of the date of first offered appointment (FOA).

Attendance report 2 (secondary end points):

- Attendance within 90 days of the date of FOA;
- Attendance within 180 days of the date the episode was opened, which is the time frame used to calculate screening uptake;
- Attendance at the date and time of the FOA.

A power calculation, using the ‘sampsi' option in STATA 12 (StataCorp, 2011) demonstrated that 20 000 women entered into the study (10 000 in each arm) would confer 84% power to detect an increase from 65 to 67% attendance as significant.

### Ethical considerations

To ensure that the women receiving the reminder letter did not feel pressurised into attending their screening appointment if they had made a decision not to attend, it was ensured, in consultation with experts and lay persons, that these letters were non-coercive in tone. There were considered no potential harms to the recipients of these letters. This study was approved by the South Central Oxford A Research Ethics Committee (application number 12/SC/0722). The trial was registered with the ISRCTN registry, number 02240458 (http://www.controlled-trials.com/ISRCTN02240458).

## Results

**Figure 1:**
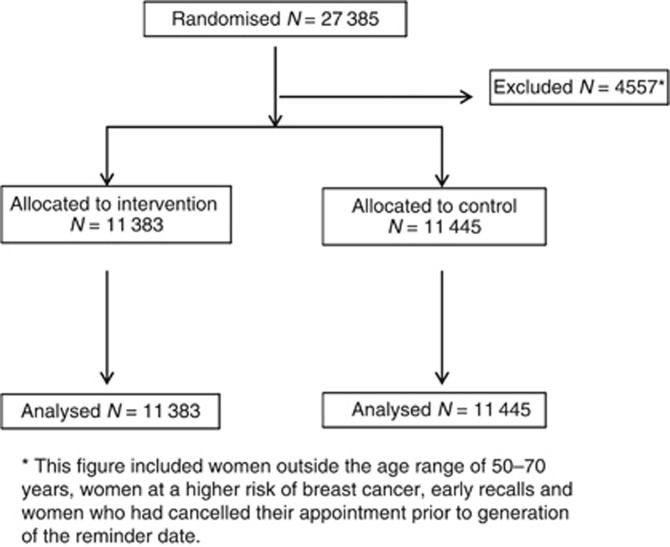
Study procedure (adapted from the CONSORT Transparent Reporting of Trials).

Overall, 27 385 women were recruited into this study. In all, 4557 women were excluded because of the reasons stated in the Materials and Methods section. This left 22 828 women for the analyses: 5619 (24.6%) from Wigan, 5726 (25.1%) from Liverpool and 11 483 (50.3%) from Bolton. A total of 11 383 (49.9%) women were randomised to receive the intervention of an invitation reminder letter and 11 445 (50.1%) to the control arm (usual invitation procedures) (Figure 1). Descriptive statistics of both groups can be found in Table 1. As expected, there was no significant difference between those randomised to receive the invitation letter compared with those who were not, for socioeconomic status, age at FOA or whether it was a woman's first (prevalent) or subsequent (incident) screen.

In total, 15 108 (66.2%) of the women attended for screening within 30 days of the FOA (Table 2). Those receiving the intervention were significantly more likely to attend compared with those who did not receive it (*P*<0.001): 7759 (68.2%) *vs* 7349 (64.2%). The OR of attending screening within 30 days of the FOA if in the intervention arm was 1.19 (1.13–1.26) compared with the control arm.

The following secondary end points were analysed:

The OR of attending for women being screened within 90 days of the FOA was 1.16 (1.09–1.23), 8141 (71.1%) in the control *vs* 8430 (74.1%) in the intervention arm attended within 90 days FOA (Table 2).
The OR (95% CI) of women attending within 180 days of the episode being opened was very similar at 1.14 (1.08–1.22), 8254 (72.1%) in the control *vs* 8511 (74.8%) in the intervention arm attended within 180 days of the episode being opened (Table 2).
In all, 10 749 (47.1%) of all women attended screening on their FOA date, that is, they did not change their appointment date. Of these, 5531 (49.9%) were in the intervention arm compared with 5218 (45.6%) in the control arm, a significant difference (*P*<0.001).

Similar effects of the intervention were observed for women aged <60 years and women aged 60 years and above who attended within 30 days of their FOA with ORs of attending of 1.19 (1.10–1.29) and 1.19 (1.10–1.30), respectively: 3861 (62.5%) in the control *vs* 4068 (66.5%) in the intervention arm for women <60 years of age and 3488 (66.2%) in the control *vs* 3691 (70.1%) in the intervention arm for women 60 years of age and above (Table 3). Analyses of 90 days after the FOA and 180 days after the episode was opened for these women supported these results.

Overall, there were 6264 out of 22 828 (27.4%) women who were offered a first (prevalent) screen and 16 564 (72.6%) who were offered a subsequent (incident) screen. Screening attendance within 30 days of FOA was significantly higher for women offered an incident *vs* a prevalent screen irrespective of whether they were randomised to the intervention or control arm (75.1% incident screen compared with 42.7% prevalent screen for attending within 30 days of FOA). The low prevalent screen uptake is owing to the fact that this includes women who have been invited before but have never attended. To take this into account, Table 4 shows the results for the prevalent round for younger women, that is, aged 50–52 years and the incident round for women 53–70 years by whether they attended the previous screen or not. For younger women in the prevalent round and for older women in the incident round who had attended their routine screen 3 years previously, attendance within 30 days of the FOA was significantly greater for those in the intervention arm compared with those in the control group. ORs (95% CI) were 1.21 (1.05–1.39) and 1.26 (1.17–1.37), respectively. Analysis of 90 and 180 days supported these results for both groups. For older women who had not attended their last routine screen 3 years previously, numbers were too small for significance, but attendance within 30 days was in the direction showing benefit to women in the intervention arm compared with the controls with an OR (95% CI) of 1.20 (0.65–2.21). For 90 and 180 days the ORs were close to 1.00, with ORs (95% CI) of 0.98 (0.54–1.79) and 0.98 (0.54–1.80), respectively.

There was significant heterogeneity of the effect of the intervention between sites (*P*=0.01), with only Bolton showing a significant increase in attendance in the intervention group within 30 days of FOA, OR (95% CI) 1.30 (1.20–1.41). Wigan and Liverpool had ORs (95% CI) of 1.10 (0.98–1.24) and 1.08 (0.97–1.21), respectively (Table 5), with both of borderline significance (*P*=0.08 and 0.1, respectively). Secondary results showed a significant effect of the intervention for Bolton for both 90 and 180 days: OR (95% CI) 1.24 (1.15–1.36) and 1.22 (1.13–1.33), respectively. Wigan showed a borderline effect for both 90 and 180 days: OR (95% CI) 1.13 (1.00–1.29) and 1.13 (0.998–1.29), respectively, and Liverpool showed a nonsignificant effect with ORs (95% CI) 1.03 (0.92–1.53) and 1.02 (0.91–1.15) for both 90 and 180 days, respectively.

Almost 30% of all the women in this study were in the lowest socioeconomic quintile and only 11.1% in the highest. 21.5%, 16.6% and 20.9% were in the second, third and fourth lowest socioeconomic quintile, respectively. Women in Liverpool were the most deprived, with 42% in the lowest socioeconomic quintile and only 2.6% in the highest. There were 31.9% of women in Bolton who were in the lowest quintile and 9.1% in the highest. In contrast, women in Wigan were more affluent, with 13.5% in the lowest quintile and 23.9% in the highest. Logistic regression analysis using the continuous variable of IMD score, where a higher score indicates lower socioeconomic status, showed that a woman was less likely to attend the lower her socioeconomic status was, regardless of study arm. The effect of the intervention did not vary by IMD score (results available from the authors).

## Discussion

This study shows a significant improvement from 72.5 to 75.1% in eventual uptake for breast screening and an improvement from 64.2 to 68.2% in attending within 30 days of FOA as a result of a postal reminder of a woman's imminent breast screening appointment. This indicates that the intervention, in addition to improving overall uptake and therefore the public health impact of the screening programme, also improves attendance at first booked appointment, conferring a reduction in appointments wasted through non-attendance and rebooking. Age, socioeconomic status and type of screen (prevalent or incident) did not significantly modify this effect.

The limitations of this study are the expedient of using odd or even SX numbers for allocation to the trial arm rather than formal randomisation, and the fact that the study took place entirely within one region, the north-west of England. As noted above, there is no systematic practice in allocation of odd or even SX numbers, and thus we submit that this does not detract from the validity of the results. The areas in which the study took place had lower uptake than the national average, thus absolute uptake rates cannot be generalised, although it is highly probable that the effect of the intervention would apply nationally.

This study lends weight to conclusions from numerous other studies that have looked at various additional interventions to increase participation in breast cancer screening. A Cochrane review concluded that active recruitment strategies including direct reminders increased participation (Bonfill Cosp *et al*, 2001). This was borne out by a more recent review incorporating other (non-breast) cancer screening programmes (Camilloni *et al*, 2013). A study in Italy of different methods of involving and informing women about mammography found that a reminder letter increased participation rate in 5360 Italian women aged 40–45 years by an average of 7.7% (Giordano *et al*, 2011). Studies in the Republic of Ireland have found increases of 17.9–29.9% with reminder letters (Hayes *et al*, 1999; Fleming *et al*, 2012). A survey of 50–60-year-old women in Ontario, Canada explored the acceptability of reminder letters for breast cancer screening. The women found them to be useful and they also appeared to influence the women's decision to undergo mammography screening (Kaczorowski *et al*, 2009).

Interestingly, for women aged 53–70 years who had not previously attended, the intervention had no effect on ultimate participation rates, but did improve attendance within 30 days of FOA. This suggests that its effect in this group is only to change the time of attendance to the FOA or near that time.

The more resource-intensive intervention of a telephone reminder has also been observed to improve participation in breast screening. In an area of East London, characterised by ethnic diversity and populations of low socioeconomic status, a telephone reminder campaign was observed to be associated with a greater increase in participation, of the order of 10% (Offman *et al*, 2014). However, the baseline participation rate was low in the area of that study, around 50%, and thus there was considerably more room for improvement. In addition, the telephone reminder came from the invitee's general practice, and so had an implicit GP endorsement. A randomised trial in Belgium found that there was a 4% increase in breast screening attendance among 3880 women aged 50–69 years who received a tailored telephone reminder compared with controls (Goelen *et al*, 2010).

Another attractive option is the mobile phone text message, often used to send reminders of dental appointments. This has the immediacy of a telephone call, but does not have the confrontational aspect or the human resource outlay. A randomised trial in the London borough of Hillingdon tested whether text messages were effective in improving attendance for women aged 47–53 years receiving their first invitation to breast screening and found that it was, with an OR of 1.26 (1.05–1.48). The OR only including those women who had a mobile phone number recorded (41%) was 1.71 (1.29–2.26) (Kerrison *et al*, 2015).

It is interesting to note that the effect of a reminder letter was greater in Bolton than in the other two sites. The effects in the other two sites were, however, in the same direction, with at least suggestive levels of significance. Additionally, Bolton recruited much larger numbers than either of the other two sites, and so had greater statistical power to find a significant result. However, it is possible that local issues may modify the effect of the intervention and these might require further investigation before implementation. It is difficult to see what might render Bolton more responsive to the intervention than the other two areas. It is more deprived than Wigan, but less so than Liverpool (all three areas are more deprived than the England average). Although Bolton has the largest ethnic diversity of the three areas, all have more than 80% white British or Irish populations. In terms of health statistics, Bolton does have more favourable results than the other two areas in terms of a number of indices, including smoking-related deaths, alcohol-related harm, obesity and physical activity (http://www.apho.org.uk/default.aspx?QN=P_HEALTH_PROFILES). It may be that populations that already have a greater degree of health consciousness are more responsive to health reminders such as that evaluated in this study.

A formal cost-effectiveness analysis is planned, which will estimate the incremental cost-effectiveness ratios for the average effect and for the range of effects observed in this trial.

It is desirable to find approaches that allow women to make an informed choice about whether to attend for screening and to make screening accessible and acceptable to maximise the attendance of those who wish to be screened. It seems logical to suggest that the intention to attend is a key concept and a strong predictor of actual attendance. Therefore, those women with this intention would benefit from practical interventions such as reminders. The postal reminder evaluated here might be practicable to implement across the NHSBSP.

## Figures and Tables

**Table 1:** Patient characteristics by randomisation status

	**No intervention, *N* (%)**	**Intervention, *N* (%)**	**Test**
	11 445 (50.1)	11 383 (49.9)	
Mean IMD score (range)^a^	27.4 (2.2–82.0)	27.4 (2.2–81.6)	Wilcoxon's rank-sum test *z*=0.01, *P*>*z*=0.99
**IMD, *N* (%)^b^**			
1st quintile	3392 (29.8)	3392 (30.0)	*χ*^2^ for trend *z*=0.33, *P*>*z*=0.74
2nd quintile	2486 (21.9)	2384 (21.1)	
3rd quintile	1878 (16.5)	1880 (16.6)	
4th quintile	2336 (20.5)	2409 (21.3)	
5th quintile	1279 (11.3)	1242 (11.0)	
Mean age at first offered appointment (years)	59.0 (50–70)	59.0 (50–70)	Wilcoxon's rank-sum test *z*=0.36, *P*>*z*=0.72
**Age group (years), *N* (%)**			
<60	6179 (54.0)	6119 (53.8)	Pearson's *χ*^2^ (1)=0.12, *P*=0.72
60+	5266 (46.0)	5264 (46.2)	
**Screen type**			
Prevalent	3110 (27.2)	3154 (27.7)	Pearson's *χ*^2^ (1)=0.82, *P*=0.37
Incident	8335 (72.8)	8229 (72.3)	
**Centre**			
Bolton	5745 (50.2)	5738 (50.4)	Pearson's *χ*^2^ (2)=0.30, *P*=0.86
Wigan	2835 (24.8)	2784 (24.5)	
Liverpool	2865 (25.0)	2861 (25.1)	

Abbreviation: IMD=index of multiple deprivation.

a Higher score=lower socioeconomic status.

b 1st quintile=lowest socioeconomic class.

**Table 2:** Attendance within 30 and 90 days^a^ and within 180 days^b^

	**Attenders (%)**			
**End point**	**No intervention**	**Intervention**	**OR (95% CI)**	**Significance**
**Attending within 30 days FOA**				
Yes	7349 (64.2)	7759 (68.2)	1.19 (1.13–1.26)	*P*<0.001
No	4096 (35.8)	3624 (31.8)		
**Attending within 90 days FOA**				
Yes	8141 (71.1)	8430 (74.1)	1.16 (1.09–1.23)	*P*<0.001
No	3304 (28.9)	2953 (25.9)		
**Attending within 180 days of episode opened**				
Yes	8254 (72.1)	8511 (74.8)	1.14 (1.08–1.22)	*P*<0.001
No	3191 (27.9)	2872 (25.2)		

Abbreviations: CI=confidence interval; FOA=first offered appointment; OR=odds ratio.

a Of the first offered appointment date.

b Of when the episode was first opened, by randomization.

**Table 3:** Results by age at randomisation

		**Attenders/total (%)**		
	**End point**	**No intervention**	**Intervention**	**OR (95% CI)**
**Age (years)**				
<60	Days from FOA			
	30	3861/6179 (62.5)	4068/6119 (66.5)	1.19 (1.10–1.29)
	90	4298/6179 (69.6)	4442/6119 (72.6)	1.16 (1.07–1.25)
	Days from episode opened			
	180	4365/6179 (70.6)	4495/6119 (73.5)	1.15 (1.06–1.25)
				
60+ years	Days from FOA			
	30	3488/5266 (66.2)	3691/5264 (70.1)	1.19 (1.10–1.30)
	90	3843/5266 (73.0)	3988/5264 (75.8)	1.15 (1.06–1.27)
	Days from episode opened			
	180	3889/5266 (73.9)	4016/5264 (76.3)	1.14 (1.04–1.25)

Abbreviations: CI=confidence interval; FOA=first offered appointment; OR=odds ratio.

**Table 4:** Results for women aged 50–52 years^a^ and 53–70 years^b^

		**Attenders/total (%)**		
	**End point**	**No intervention**	**Intervention**	**OR (95% CI)**
**screen**				
Prevalent round for women aged 50–52 years inclusive	Days from FOA			
	30	1050/1772 (59.3)	1157/1814 (63.8)	1.21 (1.05–1.39)
	90	1152/1772 (65.0)	1266/1814 (69.8)	1.24 (1.08–1.43)
	Days from episode opened			
	180	1164/1772 (65.7)	1280/1814 (70.6)	1.25 (1.08–1.44)
				
Incident round for women aged 53–70 years inclusive who previously attended	Days from FOA			
	30	5313/7157 (74.30	5590/7120 (78.5)	1.26 (1.17–1.37)
	90	5884/7157 (49.3)	6057/7120 (85.1)	1.23 (1.12–1.35)
	Days from episode opened			
	180	5964/7157 (83.3)	6106/7120 (85.8)	1.20 (1.09–1.32)
				
Incident round for women aged 53–70 years inclusive who did not previously attended	Days from FOA			
	30	36/90 (40.0)	37/83 (44.6)	1.20 (0.65–2.21)
	90	47/90 (52.2)	43/83 (47.8)	0.98 (0.54–1.79)
	Days from episode opened			
	180	48/90 (53.2)	44/83 (47.8)	0.98 (0.54–1.80)

Abbreviations: CI=confidence interval; FOA=first offered appointment; OR=odds ratio.

a Inclusive for prevalent screen.

b Inclusive for incident screen by previously did and did not attend.

**Table 5:** ORs for attending screening within 30 days of first offered appointment date by site

	**Attenders/total (%)**		
**Sites**	**No intervention**	**Intervention**	**OR (95% CI)**
Bolton	3658/5745 (63.7)	3992/5738 (69.6)	1.30 (1.20–1.41)
Wigan	1962/2835 (69.2)	1986/2784 (71.3)	1.10 (0.98–1.24)
Liverpool	1729/2865 (60.4)	1781/2861 (62.3)	1.08 (0.97–1.21)

Abbreviations: CI=confidence interval; OR=odds ratio.
