# Supplementary material for: A randomised trial of the effect of postal reminders on attendance for breast screening
Source: Br J Cancer. 2016 Jan 19;114(2):171–6. doi: 10.1038/bjc.2015.451 (PMC5154292; doi:10.1038/bjc.2015.451)
Supplement: Supplementary Information [file bjc2015451x1.docx]

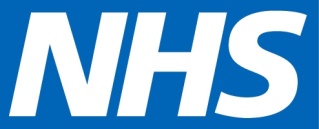
**< local letter head>**

<**Address**>

<**Date**>

<**Greeting Line**>

This is a **reminder** that your breast screening appointment is on:

**<Date Of Clinic>**

**<Appointment Time>**

<**Clinic Name On Letters**>

Screening can spot changes to your breasts which may not be visible to you. Attending screening is a good way of finding cancer early which can save lives.

It is important you attend breast screening but if the above time and date is not suitable please call us as soon as possible on **< local phone number>** to rearrange.

For information about breast screening, including what to expect at your appointment, visit [<](http://www.cancerscreening.nhs.uk)local web-page>

**Breast Screening = Early Diagnosis = Saves Lives**

Please ignore this reminder if you have cancelled or changed your appointment in the last few days.
